# Human Health Risk Assessment Applied to Rural Populations Dependent on Unregulated Drinking Water Sources: A Scoping Review

**DOI:** 10.3390/ijerph14080846

**Published:** 2017-07-28

**Authors:** Lorelei Ford, Lalita Bharadwaj, Lianne McLeod, Cheryl Waldner

**Affiliations:** 1 School of Environment and Sustainability, University of Saskatchewan, 117 Science Place, Saskatoon SK S7N 5C8, Canada; lorelei.ford@usask.ca; 2 School of Public Health, University of Saskatchewan, 107 Wiggins Road, Saskatoon SK S7N 2Z4, Canada; 3 Western College of Veterinary Medicine, University of Saskatchewan, 52 Campus Drive, Saskatoon SK S7N 5B4, Canada; lianne.mcleod@usask.ca (L.M.); cheryl.waldner@usask.ca (C.W.)

**Keywords:** risk assessment, deterministic, groundwater, holistic, probabilistic, rural population, drinking water, human health

## Abstract

Safe drinking water is a global challenge for rural populations dependent on unregulated water. A scoping review of research on human health risk assessments (HHRA) applied to this vulnerable population may be used to improve assessments applied by government and researchers. This review aims to summarize and describe the characteristics of HHRA methods, publications, and current literature gaps of HHRA studies on rural populations dependent on unregulated or unspecified drinking water. Peer-reviewed literature was systematically searched (January 2000 to May 2014) and identified at least one drinking water source as unregulated (21%) or unspecified (79%) in 100 studies. Only 7% of reviewed studies identified a rural community dependent on unregulated drinking water. Source water and hazards most frequently cited included groundwater (67%) and chemical water hazards (82%). Most HHRAs (86%) applied deterministic methods with 14% reporting probabilistic and stochastic methods. Publications increased over time with 57% set in Asia, and 47% of studies identified at least one literature gap in the areas of research, risk management, and community exposure. HHRAs applied to rural populations dependent on unregulated water are poorly represented in the literature even though almost half of the global population is rural.

## 1. Introduction

In 2015, the World Bank identified 46% (3.38 billion) of the world’s population as rural, and determined that 15% of that population lacked adequate access to water [1]. In 2000, the Millennium Declaration was signed by the United Nations to establish the Millennium Development Goal (MDG) to reduce, by half, the number of the world’s population without “sustainable access to safe drinking water” [2]. However, increased access to water does not guarantee water sources are safe for consumption, and without sufficient water testing and mitigation of drinking water risks, rural populations are vulnerable to increased health risks associated with drinking water hazards [3,4,5]. Global rural populations remain an “at risk” priority due to: exposure to unknown drinking water hazards; a lack of oversight associated with the use of unregulated water sources; a failure to mitigate known drinking water risks (e.g., avoidance or non-regulated treatment); and their vulnerability and inequality as it relates to education and financial resources to establish safe water in comparison to urban populations [2,6,7]. To support the management of the risks to rural communities and to further the field of human health risk assessment (HHRA) it is imperative to understand the research undertaken in this area. To this point, there has not been a review or summary of the research literature that provides the type and frequency of applied HHRA methods to determine the drinking water risks to rural communities dependent on unregulated source water.

Human health risk assessment has been used to quantify risk as it relates to human exposure to potential hazards since the late 1940s. With its origins in environmental risk assessment, HHRA has since evolved independently from the environmental discipline [8]. The fields of human health and environmental risk assessment have not paralleled one another in their development of integrated risk assessment despite similarities in the traditional application of methods [9]. In 2003, Bridges hypothesized that the departmental separation of human health and environment by governments; the lack of integrated risk assessment training in universities; and the requirement for communication and collaboration between disciplines are sources of resistance to the integration of human health and environmental risk assessment. Many authors [8,9,10,11] have acknowledged the need for guidelines and frameworks to facilitate integrated assessment, and there are examples that have been suggested and developed [8,12,13,14]. However, a recent publication by Wilks et al. [15] suggests the integration of environment and health risk assessments remains a challenge due to lack of agreement between “…terminology, models and methodologies across chemical categories and regulatory agencies…”. In addition to concerns regarding the implementation of integrated risk assessment, Wilks et al. [15] acknowledge that non-traditional factors such as behaviour, socio-economics, perceptions, and values could improve the determination and management of risk through a more holistic approach.

The terms *integrated* and *holistic* are inconsistently defined as noted by Bridges in 2003. Integrated risk assessment, generally, refers to the inclusion of both human health and environmental risk in one assessment [9,12,16,17], while the term *holistic* suggests a systems approach where different data types and sources influencing risk can be utilized (e.g., social, economic, perception, etc. [18,19]. Adopting a holistic approach using probabilistic and stochastic methods can benefit HHRA by allowing for the use of alternative data sources and types [9,19,20], which can increase the accuracy through the quantification of uncertainty [21]. As mentioned previously, Wilksetal [17] suggests that a holistic approach may consider economic, social, cultural, and political factors; however, they do not describe the inclusion of these factors as a data source per se. For the purpose of this scoping review, we define integrated risk assessment according to the WHO/ICPS [12] as “…a science-based approach that combines the processes of risk estimation for humans, biota, and natural resources in one assessment.” Alternatively, we suggest that a holistic risk assessment would be similar to that described by Arquette et al. [18] and include non-traditional factors, that may be gathered from qualitative data sources or multiple disciplines, in the determination of risk that is specific and relevant to the humans or environment of concern. A holistic human health risk assessment would be inherently integrated; however, an integrated risk assessment is not necessarily holistic.

Deterministic methods of HHRA have been applied to comply with structured national and international guidance documents and frameworks. Despite studies that identify the benefits of integrated risk assessment [8,9,14,17,21,22], there has not been a systematic review of the frequency of applied deterministic, integrated or holistic methods. A scoping review of recent HHRA practices may be used to inform and support the adoption and use of holistic frameworks by government and researchers. This could improve methods and quantify uncertainty, which would support effective risk communication and management [23]. This paper summarizes HHRA research used to assess human health risks associated with unregulated drinking water and describes the frequency of HHRAs applied to rural communities, the characteristics of methods and publications, and current literature gaps.

## 2. Methods

This scoping review involved a multi-disciplinary team of four researchers in the fields of water quality, human health, epidemiology, and toxicology. Analysis and writing was the responsibility of the lead (Lorelei Ford) with all team members participating in the review process, meetings, and editing. A health sciences research librarian was consulted on the selection of databases and search terms to ensure the identification of relevant studies. The framework chosen for the review was that presented by Pham et al. [24] which is based on the works of Arksey and O’Malley [25], and Levac [26]. This review utilized the first five steps of the Arksey and O’Malley [25] framework, including: identification of the research question; identification of relevant studies; study selection; charting the data; and collating, summarizing and reporting results.

### 2.1. Research Question

The research questions asked included, “What methods of HHRA have been used to determine the health risks associated with consumption of unregulated drinking water, and how often are they applied within the context of rural communities?”

### 2.2. Data Sources and Search Strategy

In January 2014, two researchers (Lorelei Ford and Lianne McLeod), with the assistance of a research librarian at the University of Saskatchewan Health Sciences Library, identified the databases, search terms, and limitations that would define the review. Search databases included ProQuest—Public Health (multidisciplinary); EMBASE − Embase + Embase Classic (biomedical, broad); MEDLINE-Ovid (biomedical, specific); Global Health (global); and Scopus (multidisciplinary, broad). These databases provided comprehensive coverage of a wide range of disciplines as they relate to human health risk assessment. Search terms included: “risk”, “risk assessment*” or “analys*”, “water”, “groundwater”, and “health”. The search terms “risk assessment”, “water” and “groundwater” were expanded to ensure inclusion given the diverse range of terminology for HHRA. The concatenated term “groundwater” was specifically included because search terms for “ground” and “water” returned fewer results. Search terms did not include “drinking water” because studies using the term were included using the search term “water”. Searches were restricted to English language publications between 1 January 2000 and 8 May 2014. The Scopus search excluded newspaper articles due to the otherwise high number of non-peer reviewed articles. Detailed search strategies are provided in Supplementary Materials.

### 2.3. Citation Management

Search results were exported to Microsoft Excel and imported to Microsoft Access (Microsoft Corporation, Redmond, WA, USA) for title and abstract relevance screening. Citation fields included: author, reference, journal, title, and abstract. Each database was independently de-duplicated and then combined. Duplicates were identified and eliminated independently by two researchers (Lorelei Ford and Lianne McLeod) and agreement confirmed.

### 2.4. Eligibility Criteria

Study selection was a two-step screening process involving a title and abstract screen. In addition to title and abstract screening methods identified by Arksey and O’Malley [25] and Pham et al. [24], abstracts were categorized, according to the inclusion criteria in Table 1, by two researchers (Lorelei Ford and Lianne McLeod) during screening to enable reliable sorting for full-text review.

Titles were included if it was clear they *were* or *could* be about risk assessment and drinking water, to minimize the potential for exclusion of relevant articles. For this scoping review, regulated water sources (e.g., municipal treatment, community treatment, or centralized water sources for cities and towns) were excluded to focus the review on unregulated water sources (e.g., private drinking water wells, raw water sources, etc.). Unspecified water sources represent a category of studies that failed to confirm the water source as unregulated and did not describe the site, hazards tested, or circumstances to suggest water was regulated. Unspecified water sources, likely unregulated, were included in analysis to identify shortfalls in reporting but excluded from descriptive statistics when specifically addressing unregulated water sources.

### 2.5. Title and Abstract Relevance Screening

Titles and abstracts were scanned independently by two researchers (Lorelei Ford and Lianne McLeod) to prevent exclusion of valid citations. Disagreements between reviewers during this scan resulted in the article’s inclusion for full-text review. A form was created in Microsoft Access to categorize the abstracts to reach consensus on meeting inclusion criteria. The title and abstract scans were completed 6 and 20 November 2014, respectively.

### 2.6. Data Characterization

Articles meeting inclusion criteria were eligible for full-text review. Themes and categories were developed and defined based on specific references and terms to ensure characterization of data was consistent. Three broad themes were developed to include HHRA characteristics, literature characteristics; and literature gaps. Categories within the human health risk assessment characteristics theme included the exposure population, exposure pathway, hazard identification, applied methods, framework used, HHRA terminology, factors and uncertainty, and outcomes specific to the application of risk assessment. Literature characteristics related to the world region in which the studies took place, publication dates, and publication sector (or field). Literature gaps, defined as any gap identified in the study by authors, generally fit into three categories including gaps in HHRA research, risk management, and community exposure. Except for a few cases, in which researchers contacted authors by email via ResearchGate (ResearchGate GmbH, Berlin, Germany), full-text articles were accessed through the University of Saskatchewan online library. Non-peer reviewed literature was eliminated from the review. If studies did not provide sufficient evidence for exclusion, they were retained for analysis and identified as “unspecified”. Prior to full-text review, all researchers independently reviewed one randomly selected article (i.e., [27]) and discussed themes, categories, and definitions as suggested by Levac [26]. Full-text review was conducted by three researchers (Lorelei Ford, Lalita Bharadwaj and Cheryl Waldner) and studies that failed to meet requirements of inclusion criteria for abstract scan and full-text review were removed from further analysis. Individual reviews were summarized and discrepancies or questionable categorizations were re-examined prior to combining results. Final categorization was completed on 30 June 2015. A detailed list of the themes and categories, including examples, and references for the full-text review, are summarized in Supplementary Materials.

### 2.7. Data Summary and Synthesis

Screening and full-text review were compiled using Microsoft Excel 2010 (Microsoft Corporation, Redmond, WA, USA). All data entries were reviewed and scanned for manual errors or incomplete entries prior to analysis. Calculation of descriptive statistics, frequencies, and percentages on nominal data was performed using Microsoft Excel 2010. Charts were designed using Tableau 9.1 (Tableau Software Inc., Seattle, WA, USA).

## 3. Results

### 3.1. Search and Selection

**Figure 1:**
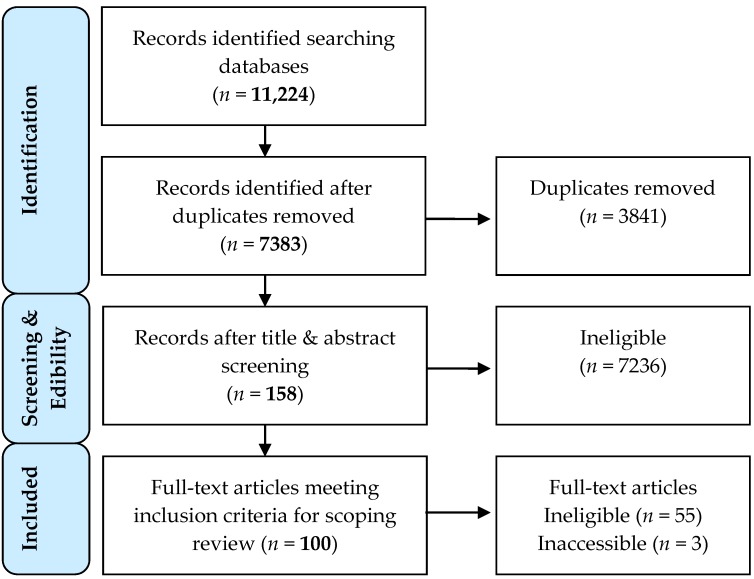
PRISMA flowchart of scoping review process. PRISMA: (Preferred Reporting Items for Systematic Reviews and Meta-Analysis).

One hundred papers met the inclusion criteria for data extraction and scoping review. A total of 7838 unique articles were found after database results were de-duplicated (Figure 1). Further title and abstract screening resulted in the selection of 158 studies for full text review; however, three articles could not be located (i.e., [28,29,30]) and the remaining 55 did not meet inclusion criteria.

### 3.2. Human Health Risk Assessment Characteristics

Table 2 provides a summary of the characteristics of applied HHRAs and categorized into exposure population, exposure pathway, hazard identification (including status of drinking water), applied method, scope, framework used, HHRA terminology, factors and uncertainty, and outcomes.

Human health risk assessments were applied to rural populations dependent on unregulated source water in only 7% (7/100) of the scoped studies (Table 2). Overall, unregulated water sources were identified in only 21% (21/100) of the studies, while the remaining (79%, 79/100) failed to specify the regulatory status but did not provide enough information to be excluded as regulated. Over half (54%, 54/100) of the geographic areas for the population were insufficiently described and could not be categorized as rural, urban, or remote.

Source water categories including ground and surface water were, not exclusively, identified in 67% (67/100) and 39% (39/100) of the reviewed studies, respectively (Table 2). Groundwater was categorized as unregulated in 14% (14/100) of the studies, which was double the percentage of surface water sources found to be unregulated (7%, 7/100). Regardless of the source water’s regulatory status, groundwater was identified as untreated in 64% (43/67) of the articles (e.g., [31,32,33,34,35]) versus only 10% (4/39) surface water. Only three studies (3%, 3/100) identified a rural population dependent on unregulated and untreated groundwater (i.e., [36,37,38]).

Drinking water hazards were identified as natural or anthropogenic chemicals in 82% (82/100) of articles reviewed (Table 2). Risks associated with bacteria, viruses, parasites, and radiological parameters were studied in 11% (11/100) of the HHRAs, exclusive of chemicals, with a small proportion (5%, 5/100) including a chemical hazard in addition to microbes, pathogens, or radiological parameters.

Receptors, defined as the specific group of people exposed to potential risk, were inconsistently described throughout the reviewed literature. Not mutually exclusive, the literature identified adult or local residents as receptors in 66% (66/100) and 41% (41/100) of the studies, respectively (Table 2). A specific age category for receptor descriptions was not defined in 39% (39/100) of the studies. Other receptor categories identified (Table 2) included: children, toddlers, teens, “(people) responsible for source water”, the “general public”, infants, “local farmers and families”, or “employees”. No studies identified receptors as First Nations, or indigenous communities. When the exposure population was described as a community, the population was delineated by a geographic area (86%, 86/100), topography (27%, 27/100), cultural or spiritual characteristics (2%, 2/100), or was unspecified (20%, 20/100). Unspecified populations were vaguely described as being in proximity to sources of pollution, source water, or hydro-geological influences.

Table 2 shows that 86% (86/100) of HHRAs applied to unregulated or unspecified drinking water were deterministic with 14% (14/100) utilizing probabilistic and/or stochastic methods in their analysis [27,39,40,41,42,43,44,45,46,47,48,49,50]. Only four studies had an integrated environmental risk in addition to human health (i.e., [50,51,52,53,54]). The USEPA risk assessment framework was applied in 75% (75/100) of the studies, while 6% (6/100) of the studies utilized the standardized international methods of the World Health Organization. Peer-reviewed, other government or non-government methods of HHRA were applied in 15% (15/100) of the studies while 12% (12/100) had no clear methodological framework.

Use of terminology describing HHRAs was inconsistent within and between studies. The term “health risk” or “health risk assessment” was used in 47% (47/100) of the scoped articles. Less frequently the terms “human risk assessment” or “human health risk assessment”, and “risk assessment” described the assessment in 25% (25/100) and 24% (24/100), respectively. Other articles (14%, 14/100) specifically described the assessments as quantitative microbial (or health) risk assessment, cancer risk, risk estimates, and hazard evaluations.

Non-traditional factors were acknowledged or applied qualitatively, by lending to the interpretation of risk, but were not quantified variables within the risk assessment. Non-traditional factors were acknowledged in 90% (90/100) of the studies, however, their qualitative application to the interpretation of risk was only 69% (69/100; Table 2). Geographical (76%, 76/100), social (23%, 23/100) and economic (13%, 13/100) factors were acknowledged most frequently. Only 5% (5/100) of studies recognized risk perception, or cultural/spiritual non-traditional factors. The “other” categories included: health variables (e.g., [55,56]), temporal influences (e.g., [33,38,55,57]), differences in water sources (e.g., [31,58,59]), effectiveness of risk management (i.e., [60]), and human behaviours or proximity to human activities (i.e., [54,61,62,63,64,65,66,67]).

Uncertainty was acknowledged at least once in 83% (83/100) of the articles, but only 20% (20/100) provided a section specifically dedicated to the discussion of uncertainty (Table 2). Quality assurance and quality control, and analytical detection limits were mentioned in 47% (47/100) and 38% (38/100) of the articles, respectively. Seasonal or environmental influences, such as changes in hazard concentrations over time, were identified in 38% (38/100) of studies. Data gaps (30%, 30/100) and sufficiency of sampling (28%, 28/100) were more frequently mentioned than the quality of historical data for use in the calculation of risk (10%, 10/100). Other sources of uncertainty were disclosed in 18% (18/100) of the articles and included: uncertainty associated with reference to supplementary material or methods (i.e., [68,69]); variation in exposure (i.e., [27,61,63,70,71,72,73]); insufficient toxicological data or guidelines (i.e., [43,64,70,74]); error in methods or their application (i.e., [27,43,59,61,72,75,76]); unknown immunity, virulence, reporting and diagnosis (i.e., [48]); and failure to consider secondary effects or multiple sources of risk (i.e., [36,62,75,77]).

### 3.3. Literature Characteristics

Table 3 provides a summary of the literature characteristics including the region(s) in which the research was conducted, and the number of studies published.

**Figure 2:**
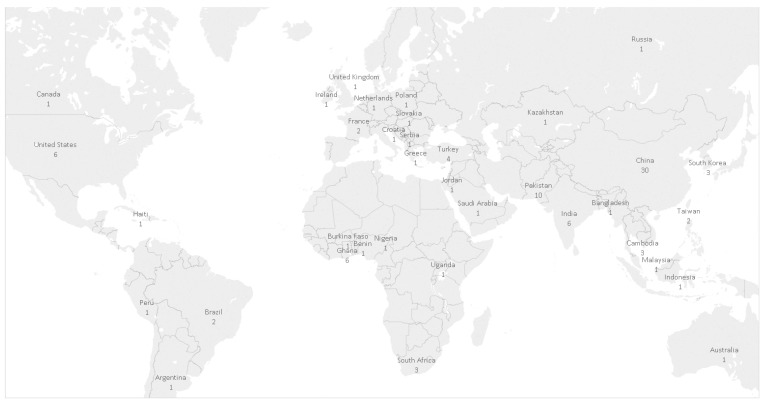
Number of scoping review studies by world region.

Most (57.4%, 58/101) of the studies were conducted in Asia and included the countries of China, Pakistan, India, and Bangladesh. All studies reported one region in which the research took place with exception of Hunter et al. [48] (e.g., France and the United Kingdom); therefore, 101 study regions were identified in the scoped literature. Figure 2 provides a visual summary of the number of studies by world region.

**Figure 3:**
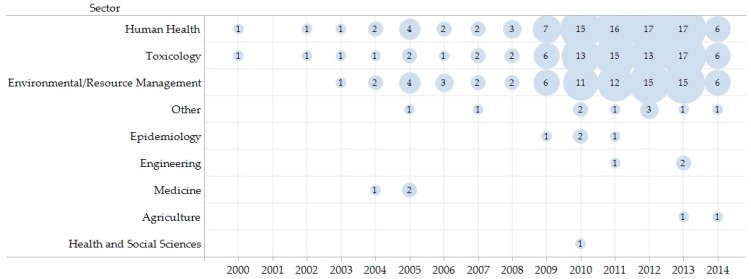
Scopin review studies by sector and year. Sectors are not mutually exclusive.

The number of articles published annually increased during the review period from January 2000 to May 2014. Twenty-five per cent (25/100) of the articles were published from 2000 to 2009, while the remaining 75% (75/100) were published in less than half that period from January 2010 to May 2014 (Table 3). The highest number of publications per year (19%, 19/100) occurred in 2013. From January 2000 to December 2013 the average publishing rate is 6.6% per year excluding studies from January to May 2014. Figure 3 provides the number of publications by sector and year where sectors are not mutually exclusive. Articles were predominately published in journals indicating a focus on human health (94%, 94/100), toxicology (81%, 81/100), and environment/resource management (79%, 79/100).

### 3.4. Literature Gaps

At least one gap in the literature was identified in 47% (47/100) of the studies. Literature gaps were not mutually exclusive and were summarized into three main categories: the research field of HHRA research (35%, 35/100), risk management gaps associated with mitigations to reduce risk (22%, 22/100), and community exposure (10%, 10/100). Table 4 provides detailed descriptions of the gaps identified in the literature and relevant studies.

## 4. Discussion

This paper provides an overview of HHRAs applied to unregulated drinking water in peer-reviewed literature and describes the frequency of their application to rural communities, the characteristics of their methodology, and gaps identified in the literature. Most of the scoped publications (79%) failed to specify the regulatory status of source water. The inclusion of literature with water sources of unknown regulatory status reveals the need to improve characterization of source water hazards in HHRA. Although 28% of applied HHRAs were identified as taking place in rural communities, only 7% clearly identified both a rural population and unregulated water source. Similarly, in a third of the articles the source water was not specifically described as raw or treated. This lack of transparency in identifying the population of concern has been previously described in a review by Pons et al. [96] of waterborne disease outbreaks in Canada and the United States, and appears to be an on-going oversight by authors reporting on risk associated with drinking water. It is essential to describe the population of concern and the regulatory status of source water utilized for drinking purposes to effectively assess the potential drinking water risks to global rural communities; to support development of appropriate risk management options; and to further research in the discipline of human health risk assessment.

The water source (i.e., ground, surface, and other) was highly reported in the studies which suggest that groundwater (67%) was the most frequent source of drinking water; however, only 14 of the studies identified the groundwater source as unregulated. Although only 21 studies identified an unregulated drinking water status, it is possible that 51 of the studies that did not specify the regulatory status but identified untreated water could be identified as unregulated. A high proportion of unregulated groundwater use would be expected given the global effort to meet the needs of increasing populations and improve accessibility of drinking water in rural and remote locations [2,96,97,98]. Information on source, treatment and regulatory status of drinking water is essential for effective use of reported data. The potential for risk is very different between treated and untreated sources. For example, treated water may pose risks associated with disinfection by-products, while raw groundwater sources may focus on naturally occurring heavy metals. The very nature of unregulated source water implies a lack of management options such as regular maintenance and monitoring. Without clear identification of drinking water supplies, and reliable information, data, and reporting, it is difficult to gauge risk and provide risk management options to rural communities.

The application of HHRA methods was largely deterministic with approximately 1 in 7 reporting the use of probabilistic or stochastic methods. Although these methods are not being utilized to integrate non-traditional factors into a holistic HHRA, more than half of the papers mentioned or qualitatively applied non-traditional factors to the interpretation of risk. For example, the most frequently acknowledged non-traditional factor was geography, which was often used to define the area associated with the hazard or to compare risk between specific areas. A shift from deterministic to probabilistic methods (which can utilize stochastic distributions) has benefits including: the quantification of uncertainty [9,99]; less dependence on animal based studies [9]; increased transparency in the process of risk assessment [99]; the potential inclusion of qualitative information [19]; and the use of vast and multiple data types [20]. In the context of this review, only four studies carried out what the WHO/ICPS [12] defined as an integrated risk assessment, and only Genthe et al. [53] further met the scoping review criteria addressing rural population consuming unregulated source water.

Holistic approaches using probabilistic risk assessment methods and decision-type networks (e.g., Bayesian Risk Assessment) that can utilize qualitative and quantitative data were not applied in the literature despite frequent acknowledgement and use of non-traditional factors to interpret risk (e.g., comparison of risk between geographical areas). The integration of qualitative data, such as behaviour, can improve risk management due to its influence on water use and exposure for rural communities [100,101]. Researchers could explore the benefits of probabilistic and stochastic methods in holistic HHRA to integrate non-traditional factors potentially influencing risk and to better characterize uncertainty [19]. For example, effective education or government programming to alter human behaviour can be used decrease exposure to hazards, rather than treating illness outcomes. Therefore, by determining how the behaviour changes the overall risk, the strategy for risk communication and management can be tailored to the receptors. Researchers continue to rely on traditional methods of HHRA despite the advances in software and data processing capability; the on-going need to improve the use of data and accuracy of risk assessment; and encouragement to use probabilistic methods by governments (i.e., [102]). Probabilistic methods in HHRA can enable more holistic risk assessments (e.g., [20]), similar to the environmental field (e.g., [103]), to assess not only multiple hazards but to include non-traditional factors that may influence risk.

The potential influence of non-traditional factors is related to uncertainty if they have an influence on the overall measure of risk [104,105,106]. Uncertainty is an important part of any risk assessment because it provides the caveats that may affect the interpretation of the risk measure. Fewer than half of the papers reported quality assurance and control within their studies. Declaration of uncertainty is fundamental to risk assessment [99] and well-established frameworks provide checklists to ensure users disclose uncertainty [107,108]. Twenty per cent (20%) of the reviewed research papers addressed uncertainty and limitations under a specific sub-heading in the article. Without full disclosure of uncertainty, it is difficult compare or assesses risk evaluations.

A significant short-coming identified in the literature was a lack of defined exposure populations considered at risk. This can be improved when thorough descriptions of receptors are provided [109]. For example, age groupings for receptors and terms such as “rural” and “urban” should be defined with geographic area for better characterization of risk. Adult receptors were frequently chosen to represent communities while sub-groups or sensitive populations were less frequently identified. The scoped studies had limited demographic representation of the receptors and considered only a single route of exposure. Despite the perceived need for inclusion of all exposure pathways as identified in the literature [82,83,84], the oral pathway of exposure was most frequently assessed.

Communities were often defined by a geographic or topographic area, implying a natural link between groundwater hazards and the physical environment, notably the geology or land-use. However, a geological approach including the interpretation of hydro-geology could be more relevant when associations between geology and hazards are required (e.g., [110]). Typically, receptors in studies were vaguely identified as “local residents”, “general public”; “local farmers and their families”; and “individuals responsible for their source water”. Related to the need to better describe the receptors, researchers identified gaps in community exposure including the necessity to address additional receptor groups or communities to improve aspects of risk assessment or management options. Clearly defining the receptors and communities in the human health risk assessment further improves the research, allowing future research to build on the knowledge associated with the characteristics of similar receptors.

Studies most frequently identified natural and anthropogenic chemical hazards. The focus of the studies on chemicals, versus bacteriological water quality parameters, suggests that the unspecified and untreated (51%) water sources in the studies may largely be unregulated groundwater; however, we are unable to confirm this. Interestingly, bacteria, pathogens, and radiological parameters were infrequently included in studies despite their presence in surface and groundwater [111,112]. Thus, future research considering risk associated with chemical, radiological, and microbiological parameters may provide a more comprehensive measure of risk for communities dependent on unregulated source water. Source water and specific hazards were generally well defined in the scoped studies; however, risk management or mitigation would benefit from comprehensive characterization of hazards and receptors including: mixed chemical or hazard exposures; geographical/geological influences; social/societal factors; and limitations and uncertainties associated with all aspects of HHRA.

The relative frequency of HHRA research on unregulated or unspecified drinking water is variable globally and is primarily focused in Asia. Since 2000, the majority of HHRA studies have taken place in countries with large numbers of rural residents without improved drinking water sources and high exposures to natural and anthropogenic water quality hazards (e.g., China and India [4,113]. Conversely, there is an absence of HHRA studies conducted in more developed regions with known drinking water hazards (e.g., North America). Villanueva et al. [112] suggest that assessing drinking water exposure is a challenge due to insufficient information on hazards and exposure. Therefore, global rural populations reliant on unregulated drinking water, regardless of regional socio-economic status, may be at increased health risk due to a mistaken perception that hazards are low. Alternatively, underutilization of safe unregulated drinking water is a missed opportunity to provide sustainable water to rural populations. Considering the development status of countries, a developed region (e.g., North America) would have the resources required to drastically reduce the risks to their population reliant on unregulated drinking water, and the research and risk management strategies carried out may provide insight into the larger global challenge of improving access to safe drinking water.

Research publications focusing on unregulated or unspecified drinking water increased from 2000 to 2014. For the scope of this review, publications prior to 2000 were not included because the literature is dated and data analysis methods have since advanced with mainstream use of computers for analysis [103,114]. Regardless, this review determined an approximate 7% annual publication rate, which is similar to the global exponentially increasing annual publication rate of approximately 8% from 1980 to 2012 [115]. In addition to increased publishing, the Millennium Declaration was established in 2000 by the Member States of the United Nations leading to the Millennium Development Goals and United Nations initiatives which have focused on improving access to safe drinking water and sanitation internationally [2]. These programs “gained momentum in the 2000s” [116], which may have created increased funding opportunities for drinking water research in countries with large rural populations lacking access to safe and sustainable drinking water. If these global initiatives are influencing publications, drinking water research, particularly in undeveloped countries and vulnerable communities, should to continue in increase the wake of international initiatives such as the World Health Organization’s Water Quality and Health Strategy 2013–2020 [3].

Publications on drinking water quality and human health best fit into journals addressing the interrelationship between disciplines focused on human health, risk assessment, and the environment. Researchers conducting risk assessments on human health should use the full description human health risk assessment instead of variations that introduce ambiguity. Human health risk assessments are defined by the US EPA [117] as “…the process to estimate the nature and probability of adverse health effects in humans who may be exposed to chemicals in contaminated environmental media, now or in the future”. Use of standardized terminology in title and abstract would ensure risk assessments with human subjects are easily identified during literature searches. Increased consistency in use of terminology, in addition to improvements already discussed (i.e., need to better characterize the hazards and receptors), would improve the clarity and transparency of applied HHRAs.

Fewer than 50% of studies identified gaps in the literature. Risk assessment short-comings were identified more frequently than gaps in risk management or community exposure. Risk assessment gaps often included the need for increased epidemiological and toxicological data, in an effort to understand the toxicological effects when exposed to chemical mixtures through multiple pathways. Risk management gaps, identified by researchers, expressed a similar need for increased data and monitoring, and improved evaluations of exposure. In addition, risk management gaps highlighted the desire by researchers to have specific national or regional HHRAs. We can summarize the gaps identified by researchers in the field of HHRA to say that overall they require: increased data collection and monitoring as well as strong integration with research fields that support HHRA (e.g., toxicology and epidemiology); the determination of risk by way of standardized methods and guides that improve accuracy and account for uncertainty; community-based research approaches that consider how the data and results can be used to support on-going drinking water and management; and improved communication and involvement with communities to ensure the outcome of HHRA studies are specific and relevant as it relates to the receptors and their exposure.

In addition to risk assessment and management gaps identified in the scoped studies, the need for risk characterization specific to communities has been recognized by researchers. Consideration of non-traditional factors (e.g., quality of life, socioeconomic, and political) has been suggested and supports the need to determine how these factors may influence risk. The importance of HHRA to protect community health requires transparency and diligent data collection, analysis, and reporting. This could be achieved through equal partnerships with communities and would be beneficial, ethical and in practice with a community based participatory research approach where both the researchers and community would benefit [104,118]. In the context of HHRA, it is ideal to meet the goals of research and management for applied research that benefits both academia and communities.

## 5. Strengths and Limitations

This scoping review was carried out with a systematic approach. Inclusion of five databases, each varied in breadth and depth, ensured the necessary coverage required for this review. The multidisciplinary team and frequent communication provided a balanced process and facilitated consensus through screening and full-text review, thus, eliminating the need for reliability statistics. A professional librarian guided initial database searches decreasing the likelihood of bias or error associated with attaining citations relevant for review. Abstract categorization assisted in development of inclusion or exclusion criteria for full-text review while allowing the team to become familiar with the literature as recommended by Daudt et al. [119]. Consistent with Pham et al. [24], full-text reviews did not include qualitative or quantitative assessment of research quality. Research team meetings at each step through the scoping process were necessary to integrate advice from the team, and maintain effective communication [119].

The possibility exists that relevant articles were excluded. Ending the search in May 2014 limited interpretations of publication trends up to publication. Despite mutually established and well-defined definitions for charting, the full-text review between researchers is subject to interpretation error. Exclusion of regulated water sources limited our ability to compare the characteristics of the scoped studies to regulated sources; however, the focus of this scoping review was to determine the characteristics associated with HHRA studies focused on unregulated source water.

## 6. Conclusions

A summary of the HHRA literature and methods applied to populations dependent on unregulated or unspecified drinking water sources is provided. This review reveals a lack of HHRA research dedicated to rural populations dependent on unregulated source waters in spite of the global concern regarding access to safe drinking water. The majority of the scoped HHRAs were applied in countries of proportionally high rural populations globally, of which a large proportion of water is unregulated and untreated. Insufficiently defined and poorly disclosed risk assessments decrease the usefulness of the research when attempting to gather vital information on exposure populations, water sources, and hazards to further this area of study or manage risk. The field of HHRA may be delayed in the adoption of methods that allow for the inclusion of various data types and the quantification of uncertainty for a holistic approach. It is essential that literature gaps identified by researchers and summarized herein are used to inform the future direction of research and management on unregulated drinking water for the world’s rural populations. Furthermore, the adoption of community-based participatory approaches, where possible, will provide the information necessary to support risk management decision-making and improve the health of communities.

### Recommendations

Global rural populations face potential health risks related to water quality hazards associated with unregulated source water. Evolution and improvement in the approach and application of HHRA methods are necessary for a better understanding of the human health risks, and improved risk communication and management in rural populations. Recommendations for researchers, based on a summary of studies in the field of HHRA on unregulated and unspecified source waters, are as follows:

- Components of the HHRA (e.g., exposure population, source water, hazards, etc.) should be adequately described to improve the detection of potential relevant literature upon title and abstract searches, and the quality of research reporting. Consistent use of terminology and reporting associated with standardized HHRA frameworks is essential. Uncertainty and limitations should be clearly presented to allow for appropriate interpretation of the research.
- A holistic approach to HHRA should be considered when non-traditional factors are suspected of influencing the human health risk. This can be accomplished with alternative methods of risk assessment (e.g., Bayesian risk assessment) to characterize non-traditional factors and their influence on the human health risks. Gaps in the literature identify the need to consider the effects and uncertainty associated with non-traditional factors with respect to multiple hazards, exposures and pathways.
- Identification of gaps in research, management, community, and risk assessments is a necessary component of HHRA. Recognition of gaps in these areas drives research forward, paving the way for new research to better inform future approaches, frameworks, and decision-making.

## Figures and Tables

**Table 1:** Scoping review inclusion criteria to identify human health risk assessments applied to unregulated or unspecified drinking water.

Inclusion Criteria
Peer-reviewed
Identified applied HHRA
Identified water use for human consumption
Identified the water source as unregulated or unspecified *^a^*

*^a^* Professional judgement and consensus was used to categorize studies that did not identify the water source as unregulated but provided evidence that the source water was not regulated.

**Table 2:** Human health risk assessment characteristics from scoping review literature (*n* = 100).

Characteristic	Number (*n* = 100)	Percentage (%)
Exposure Population		
*Geographic Area of Population*		
Rural (rural and unregulated)	28 (7)	28 (7)
Urban (urban and rural)	16 (4)	16 (4)
Remote (remote and rural)	2 (0)	2 (0)
Unspecified	54	54
*Community ^a^*		
Geography	86	86
Topography	27	27
Cultural/Spiritual	2	2
Unspecified	20	20
*Receptors ^a^*		
Adults	66	66
Local Residents	41	41
Child	31	31
Toddler	15	15
Teen	15	15
Responsible for source water	13	13
Seniors	11	11
General Public	10	10
Infants	10	10
Local Farmers and Families	5	5
Employees	2	2
First Nation/Indigenous	0	0
Age categories not defined	39	39
Other (e.g., gender, visitors, etc.)	6	6
Unspecified	8	8
Exposure Pathway *^a^*		
Oral	100	100
Dermal	23	23
Inhalation	4	4
Hazard Identification		
*Status of drinking water*		
Unregulated (unregulated and untreated)	21 (14)	21 (14)
Unspecified (unspecified and untreated)	79 (51)	79 (51)
*Source of drinking water ^a^*		
Groundwater (unregulated groundwater)	67 (14)	67 (14)
Surface water (unregulated surface water)	39 (7)	39 (7)
Other (e.g., bottled, rain, cistern, etc.)	21	21
Unspecified	5	5
*Type of drinking water*		
Untreated	56	56
Untreated and Treated	9	9
Unspecified	35	35
*Hazard in drinking water*		
Anthropogenic chemical	35	35
Natural chemical	22	22
Anthropogenic and natural chemical	25	25
Microbiological/Pathogen (microbiological/pathogen and chemical)	10 (2)	10 (2)
Radiological (radiological and chemical)	1 (3)	1 (3)
Unspecified	7	7
At least two hazards identified	5	5
*Data source ^a^*		
Source water sampled	96	96
Historical data	13	13
Predicted/Extrapolated	11	11
Biomarkers (i.e., hair samples)	3	3
Unspecified	2	2
Applied Method		
Deterministic	86	86
Probabilistic/Stochastic	9	9
Deterministic and Probabilistic/Stochastic	5	5
Scope *^a^*		
Human Health Risk Assessment	100	100
Integrated (human and environmental)	4	4
Holistic (integration of non-traditional data)	0	0
Framework Used *^a^*		
US EPA	75	75
World Health Organization	6	6
Other (i.e., studies, government)	15	15
Unspecified	12	12
HHRA Terminology		
Health (risk) Assessment	47	47
Human Health Risk Assessment	25	25
Risk Assessment	24	24
Other (e.g., cancer risk, risk estimate, etc.)	14	14
Factors and Uncertainty		
*Non-Traditional Factors acknowledged ^a^*		
At least one non-traditional factor	90	90
Geography	76	76
Social	23	23
Economic	13	13
Risk Perception	3	3
Cultural/Spiritual	2	2
Other (e.g., behaviours, additional risks, temporal effects, etc.)	22	22
*Non-Traditional Factors applied ^a^*		
At least one non-traditional factor	69	69
Geography	56	56
Social	4	4
Economic	2	2
Risk Perception	1	1
Cultural/Spiritual	1	1
Other (e.g., behaviours, additional risks, temporal effects, etc.)	16	16
*Uncertainty acknowledged ^a^*		
At least one uncertainty acknowledged	83	83
Dedicated section to uncertainty	20	20
Quality Assurance/Quality Control	47	47
Analytical detection limits	38	38
Seasonal/Environment	38	38
Data gaps	30	30
Sufficiency of sampling	28	28
Quality of historical data	10	10
Other (e.g., exposures, toxicological factors, effects of unknown variables, etc.)	18	18
Outcomes		
*Result ^a^*		
Exposure Assessment	96	96
Hazard Assessment	95	95
Hazard Quotient/Index	81	81
Epidemiological Assessment	4	4
Other (i.e., quantitative microbial risk assessment and cancer risk)	27	27
*Conclusion by Authors*		
Quantitative	94	94
Quantitative and Qualitative	4	4
Qualitative	2	2

***^a^*** not mutually exclusive.

**Table 3:** Literature characteristics from scoping review (*n* = 100).

Characteristic		
**World Region**	**Number (*n* = 101 *^a^*)**	**Percentage (%)**
Asia	58	57.4
West Africa	9	8.9
Europe	7	6.9
European Union	8	7.9
North America	7	6.9
South America	4	4.0
South Africa	3	3.0
Middle East	2	2.0
Caribbean	1	1.0
East Africa	1	1.0
Oceania	1	1.0
**Publication Year**	**Number (*n* = 100)**	**Percentage (%)**
January 2010–May 2014	75	75
January 2005–December 2009	20	20
January 2000–December 2004	5	5

***^a^*** not mutually exclusive, one study took place in two regions.

**Table 4:** Description and references for research, management, and community gaps identified in the scoping review literature (*n* = 67).

Gap Description	References
*Research in HHRA*	
Use of biomonitoring	[78]
Improved methods or application	[48,61,72,79,80]
Sources of uncertainty	[37,47]
Determining temporal exposures	[81]
Determining future exposures	[42,74]
Considering all pathways of exposure	[41,50,82,83,84,85]
Exposure to additional hazard sources	[41,63,73,86]
Exposure to mixtures	[34,53,58,83,87]
Guides to direct researchers	[88]
Gather more epidemiological evidence and toxicological data	[37,44,49,64,65,73,79,89]
*Risk Management*	
Collect data to inform management	[49,60,64,71]
Knowledge of geochemistry and aquifers	[89,90]
Monitoring	[58,79]
Evaluation of exposures	[54,59,86]
Establish national/regional HHRAs	[39,61,77,91]
Standardize methods for mixtures	[47]
Standardize regulations	[57,92]
Improved communication, response and determination of risk	[53,62,64,72]
*Community Exposure*	
Inclusion of specific community (i.e., sensitive community members)	[42]
Isolate risks specific to communities	[48,55,93]
Consider quality of life, socioeconomic, and political factors	[75,90,94]
Improve community involvement, engagement, education, and risk management	[49,53,95]

## Supplementary Materials

The following are available online at www.mdpi.com/1660-4601/14/8/846/s1, Database Search Terms and Results.
