# Supplementary material for: Human Health Risk Assessment Applied to Rural Populations Dependent on Unregulated Drinking Water Sources: A Scoping Review
Source: Int J Environ Res Public Health. 2017 Jul 28;14(8):846. doi: 10.3390/ijerph14080846 (PMC5580550; doi:10.3390/ijerph14080846)
Supplement: Supplementary file 1 [file ijerph-14-00846-s001.pdf]

**Supplemental Materials**

Database Search Terms and Results

Search History – May 8, 2014
Database: **Ovid MEDLINE**(R) <1946 to April Week 5 2014>
Search Strategy:
--------------------------------------------------------------------------------
1 (risk adj2 (assessment* or analys*)).mp. [mp=title, abstract, original title, name of substance word, subject heading word, keyword heading word, protocol supplementary concept word, rare disease supplementary concept word, unique identifier] (199988)
2 exp Risk Assessment/ or risk assessment.mp. (189599)
3 1 or 2 (201925)
4 water.mp. or exp Water/ (562529)
5 groundwater.mp. or exp Groundwater/ (10264)
6 4 or 5 (564267)
7 exp Health/ or health.mp. (1835151)
8 3 and 6 and 7 (2603)
9 limit 8 to (english language and yr="2000 -Current") (2218)
***************************
Database: Ovid MEDLINE(R) <1946 to April Week 5 2014>
Search Strategy:
--------------------------------------------------------------------------------
1 (risk adj2 (assessment* or analys*)).mp. [mp=title, abstract, original title, name of substance word, subject heading word, keyword heading word, protocol supplementary concept word, rare disease supplementary concept word, unique identifier] (199988)
2 exp Risk Assessment/ or risk assesment.mp. (175843)
3 1 or 2 (201926)
4 water.mp. or exp Water/ (562529)
5 groundwater.mp. or exp Groundwater/ (10264)
6 4 or 5 (564267)
7 exp Health/ or health.mp. (1835151)
8 3 and 6 and 7 (2603)
9 limit 8 to (english language and yr="2000 -Current") (2218)
***************************
Database: **Embase Classic+Embase** <1947 to 2014 May 07>
Search Strategy:
--------------------------------------------------------------------------------
1 (risk adj2 (assessment* or analys*)).mp. [mp=title, abstract, subject headings, heading word, drug trade name, original title, device manufacturer, drug manufacturer, device trade name, keyword] (394871)
2 risk assessment.mp. or exp risk assessment/ (343254)
3 1 or 2 (394871)
4 water.mp. or exp water/ (823914)
5 groundwater.mp. or exp ground water/ (21259)
6 4 or 5 (825148)
7 health.mp. or exp health/ (2676374)

8 3 and 6 and 7 (4358)
9 limit 8 to (english language and yr="2000 -Current") (3509)
**************************

Database: **Global Health**
Search Strategy:
--------------------------------------------------------------------------------
1 (risk adj2 (assessment* or analys*)).mp. [mp=abstract, title, original title, broad terms, heading words] (26366)
2 risk assessment.mp. or exp risk assessment/ (22755)
3 1 or 2 (26366)
4 exp water/ or water.mp. (81998)
5 groundwater.mp. or exp groundwater/ (3695)
6 4 or 5 (82096)
7 health.mp. or exp health/ (276768)
8 3 and 6 and 7 (1811)
9 limit 8 to (english language and yr="2000 -Current") (1631)
**************************

**Scopus**
Search Strategy from ProQuest
May 08 2014 15:11
Set#
Searched for
Databases
Results
S1
all(risk NEAR/2 assessment* OR risk NEAR/2 analys*) AND all((water OR groundwater)) AND all(health)
ProQuest Public Health
2590°
S2
(all(risk NEAR/2 assessment* OR risk NEAR/2 analys*) AND all((water OR groundwater)) AND all(health)) AND la.exact("English") AND pd(>20000101)
ProQuest Public Health
2538°
S3
((all(risk NEAR/2 assessment* OR risk NEAR/2 analys*) AND all((water OR groundwater)) AND all(health)) AND la.exact("English")) NOT stype.exact("Newspapers") AND pd(>20000101)
ProQuest Public Health
2105°
° Duplicates are removed from your search and from your result count.

NOTE: When proquest search run, the numbers come out differently. However, once the last page of results is loaded, the final numbers to change to those above and the export contains 2105 records. The initial results are shown below for completeness:
Set#
Searched for
Databases
Results

S5
(all(risk NEAR/2 assessment* OR risk NEAR/2 analys*) AND all((water OR groundwater)) AND all(health)) NOT stype.exact("Newspapers") AND pd(>20000101)
ProQuest Public Health
2175

**Full-Text Review Categorization**

| THEMES | CATEGORY | DEFINITION/EXAMPLE (if applicable) |
|---|---|---|
| **Publication Type** (choose one) | Journal | Peer reviewed journal |
| | Conference Paper/Proceeding | Conference document not published |
| | Thesis | Masters/PhD |
| | Non-peer reviewed article | Government, public document, opinion paper, etc. |
| | Other (describe) | Other category of publication |
| **What is the publication year?** | Year published | Year of publication |
| **Does the journal/article fit into one of these categories?** (choose all that apply) | Human Health, Health and Social Sciences, Social Sciences, Toxicology, Epidemiology, Agriculture, Engineering, Medicine, Environmental/Resource Management | Based on journal title, scope of journal, and/or content of the paper |
| | Unspecified | Unable to determine the research category |
| | Other (describe) | Other research field |
| **What is the application of the HHRA?** (choose all that apply) | Hypothetical/Theoretical | Method paper, randomly generated data, etc. |
| | Observational/Field study | Field data is collected or historical data used in 'real life' context |
| | Unspecified | Unable to determine the application |
| | Other (describe) | Other application of the HHRA |
| **What is the scope of the HHRA?** (choose all that apply) | Integrated Risk Assessment (wide scope) | Ecological & human assessment of risk which may include socio-economic components (Bridges 2003; Sekizawa & Tanabe 2005; WHO/IPCS 2001) |
| | Human Health Risk Assessment | Only human health risk assessment conducted |
| | Holistic | Considers non-traditional factors that may influence overall risk; includes non-traditional data integration (Arquette *et al.* 2002; Bridges 2003; Serre *et al.* 2003). Does not include the mention of non-traditional factors or interpretation of risk relative to non-traditional data but rather data that contributes quantitatively to the overall determination of risk. |
| | Other (describe) | Other risk assessment scope was used |
| **How is the study described by the authors?** (choose all that apply) | Human Health Risk Assessment | "…is the process to estimate the nature and probability of adverse health effects in humans who may be exposed to chemicals in contaminated environmental media, now or in the future." (United States Environmental Protection Agency (US EPA 2015) |

| | | |
|---|---|---|
| | Risk Assessment | "The probabilities and consequences of adverse events are assumed to be produced by physical and natural processes in ways that can be objectively quantified by risk assessment." (Slovic 1999). |
| | Health (Risk) Assessment | Risk assessment as defined by Ware (1987) with the broad scope of 'health' and all of its dimensions as identified by Ware (1987) - physical, mental, social function, role function, general health perceptions but more than absence of disease but "presence of well-being" (Slovic 1999; Ware 1987). |
| | Not Reported | Authors don't describe the study in any terms |
| | Other (describe) | Other study description |
| **What method of HHRA was used?** (choose one) | Stochastic/Probabilistic | "Risk assessment that uses probability distributions to characterize variability or uncertainty in risk estimates with the outcome described as a probability distribution rather than a single number" (US EPA 2001). Chowdhury *et al.* (2009) provide examples of methods. |
| | Traditional/Deterministic | Outcomes described with a single number (Health Canada 2010) |
| | Both | Both probabilistic/stochastic and deterministic methods used |
| | Unspecified | Unable to identify the method used |
| | Other (describe) | Other method of HHRA used |
| **Was a standard method used?** (choose all that apply) | Health Canada, US EPA, WHO | Standard national or international HHRA method |
| | Unspecified | Unable to determine method used |
| | Other (describe) | Other method referenced |
| **Geographic Location** | Country | State the country |
| | Undetermined | Unable to identify the country in which the research was conducted |
| **What is the drinking water source?** (choose all that apply) | Ground | Well of any type (e.g. shallow, deep, GUDI, hand-dug, drilled, bored, etc.) |
| | Surface | Lakes, rivers, streams, dugouts |
| | Rain collection | e.g. Roof top |
| | Cistern | Water hauled from any of the above sources |
| | Bottled | e.g. commercial or regulated bottled water (i.e. bottled water from a government or private treatment facility) |
| | Undetermined | Unable to identify the water source |
| | Other (describe) | Other drinking water source |
| **What is the drinking water type?** (choose all that apply) | Treated | Subject to regulated treatment |
| | Not-Treated | Private or unregulated/unknown treatment |
| | Unspecified | Cannot identify if source is treated or not |
| | Other (describe) | Other drinking water type |
| **What data informed the** | Water source tested | As outlined in Health Canada's Guidance on peer review of HHRA for federal contaminated sites in |

| | | |
|---|---|---|
| **risk assessment?**<br>(choose all that apply) | | Canada (Health Canada 2010b). |
| | Proxy tested | e.g. bio-indicators |
| | Predicted/extrapolated | Prediction modeling or extrapolation |
| | Based on historical data | Not based on current data but pre-existing information |
| | Unspecified | Cannot identify data type |
| | Other (describe) | Other data source |
| **How is the community defined?**<br>(choose all that apply) | Cultural/Spiritual | FN, Aboriginal, Indigenous, language, ethnicity |
| | Geographic | Country, city, town, province, etc. |
| | Topographic | Watershed |
| | Unspecified | Unable to identify the community |
| | Other (describe) | Other definition for the community |
| **What is the population of concern?**<br>(choose all that apply) | Urban | As defined by the study and the country in which it was conducted. This is the approach the United Nations takes and the World Bank defines 'rural' when comparing different countries (United Nations 2015). |
| | Rural | Responsible for establishing source water, not receiving centralized, distributed, treated, and regulated water (e.g. farms, villages, hamlets, private well owners, etc). |
| | Remote | Geographically isolated or too far from urban centres to receive treated, regulated, distributed water. |
| | Both | Both urban and rural communities studied |
| | Unspecified/Undefined | Unable to determine or define the population the population accurately the way it is described by the authors |
| | Other (describe) | Other description of the population |
| **What are the hazards identified?**<br>(choose all that apply)<br>*do not interpret, only answer with reported info | Chemical (natural) | e.g. associated with natural geological characteristics to which the water is exposed |
| | Chemical (anthropogenic) | e.g. human induced, agricultural, industrial, etc. |
| | Microbiological/Pathogen | bacteria, protozoans, viruses |
| | Radiation | e.g. radon, uranium |
| | Undefined | Unable to determine the hazard |
| | Other (describe) | Other hazard identified |
| **Who are the receptors?**<br>(choose all that apply) | Responsible for Source Water | Receptor is responsible for point of use water quality |
| | First Nations/Aboriginals | Native/Indigenous populations |
| | Infants, toddler, child, teen, adults, or senior | Age categories or as described in the study |
| | General Public | Paper states or describes the general population without distinguishing any age group in particular |

| | | |
|---|---|---|
| | Local Residents | People in the area that may be exposed to the hazard |
| | Local Farmers and their families | Specifically described as farmers and/or their families |
| | Employees | People exposed through work place |
| | **Any of the above without age identified?** | Note if any of the above did not have the specific age or age category defined |
| | Undefined | Unable to determine the receptors |
| | Other (describe) | Other receptor identified in the study |
| **What are the exposure pathways?** (choose all that apply) | Oral, dermal, inhalation | Exposure pathways as described by Health Canada (Health Canada 2010b) |
| | Undefined | Unable to determine exposure pathway |
| **Was uncertainty acknowledged?** (choose all that apply) *was it at least discussed | Sufficiency of sampling, analytical detection limits, data gaps, QA/QC, seasonal/environmental factors | (Health Canada 2010a) identifies these areas of potential uncertainty for discussion. |
| | Quality of historical use information to identify chemicals of potential concern | Relevant if exposure was determined using estimated or historical data. |
| | **Was there a section addressing uncertainty?** | An explicit section of the paper was dedicated to addressing uncertainty associated with the risk assessment. |
| | Other (describe) | Other source of uncertainty identified |
| **What other factors were acknowledged?** (choose all that apply) *discussion only | Risk perception | Perception of water or risk associated with any aspect of drinking water |
| | Economic | e.g. income levels, etc. |
| | Social | e.g. education, gender, etc. |
| | Cultural/Spiritual | e.g. homelands, historical use, generational, etc. |
| | Undefined | Unable to identify other factors acknowledged in the risk assessment |
| | **Geography** | Geography is mentioned as influencing exposure to hazards or identifying receptors |
| | Other (describe) | Other factor acknowledged in the risk assessment |
| **What other factors were applied in the RA?** (choose all that apply) *is represented by data | Risk perception | Perception of water or risk associated with any aspect of drinking water |
| | Economic | See Economic – What other factors were acknowledged? |
| | Social | See Education – What other factors were acknowledged? |
| | Cultural/Spiritual | See Cultural/Spiritual – What other factors were acknowledged? |

| | | |
|---|---|---|
| that is included in risk assessment analysis | **Geography** | Geography data is used to determine areas of increased risk or comparison of regions |
| | Undefined | Unable to determine if a factor was applied to the risk assessment |
| | Other (describe) | Other factor applied in the risk assessment |
| **What were the results of the assessment?** (choose all that apply) | Exposure assessment, hazard/toxicology assessment, hazard quotient | As outlined in HC Guidance on peer review of HHRA for federal contaminated sites in Canada (Health Canada 2010b). |
| | Epidemiological assessment/analysis | Use of epidemiological studies in the evaluation/setting of microbiological guidelines for recreational water, wastewater re-use, and drinking water. As defined by Blumenthal *et al.* (2001) not Ryan (Ryan 2003) in which epidemiological information informs a full risk assessment. |
| | Qualitative assessment | Differs from quantitative because conclusions are based on 'hazard qualitative description and potency' not DNELs, and risk characterization is justified not calculated (European Chemicals Agency 2012). |
| | Other (describe) | Other result was provided |
| **Did the journal/article conclude the risk assessment?** (choose one) | Yes, quantitatively. | Quantitative result - has a quantified result stating there is a risk |
| | Yes, qualitatively. | Qualitative result - has a description identifying a risk. |
| | Yes, both quantitative & qualitative | Both qualitative and quantitative conclusions were made |
| | No | No conclusion was made by the authors |
| | Undefined | Cannot determine if there is a conclusion or not |
| | Other (describe) | Other conclusion was provided |
| **What gaps in the literature are identified?** | Literature gaps | List gaps in research as identified by the authors |
| | Describe literature gaps | |

**References**


Arquette M., Cole M., Cook K., LaFrance B., Peters M., Ransom J., Sargent E., Smoke V. & Stairs A. 2002 Holistic risk-based environmental decision making: a Native perspective. *Environmental Health Perspectives*, **110**(2):259–264. doi:10.1289/ehp.02110s2259

Blumenthal U., Fleisher J., Esrey S. & Peasey A. 2001 Epidemiology: a tool for the assessment of risk. In: *Water Quality: Guidelines, Standards and Health*, International Water Association Publishing, London, United Kingdom, pp. 135–160.

Bridges J. 2003 Human health and environmental risk assessment: the need for a more harmonised and integrated approach. *Chemosphere,* **52**(9):1347–51. doi:10.1016/S0045-6535(03)00469-7

Chowdhury S., Champagne P. & McLellan P. 2009 Uncertainty characterization approaches for risk assessment of DBPs in drinking water: a review. *Journal of Environmental Management*, **90**(5):1680–1691. doi:10.1016/j.jenvman.2008.12.014

European Chemicals Agency. 2012 *How to undertake a qualitative human health assessment and document it in a chemical safety report,* Practical Guide 15/ECHA-12-B-49-EN, European Chemicals Agency, Helsinki, Finland.

Health Canada. 2010a. Guidance on human health preliminary quantitative risk assessment (PQRA), Version 2.0. Part I. HEALTH CANADA.

Health Canada. 2010b *Federal Contaminated Site Risk Assessment in Canada, Part III: Guidance on peer review of human health risk assessments for Federal contaminates sites in Canada*, Version 2.0, Health Canada, Ottawa, Canada.

Ryan L. 2003 Epidemiologically based environmental risk assessment. *Statistical Science*, **18**(4):466–48. doi:10.1214/ss/1081443230

Sekizawa J. & Tanabe S. 2005 A comparison between integrated risk assessment and classical health/environmental assessment: emerging beneficial properties. *Toxicology and Applied Pharmacology*, **207**(2):617–22. doi:10.1016/j.taap.2005.01.047

Serre M., Kolovos A., Christakos G. & Modis K. 2003 An application of the holistochastic human exposure methodology to naturally occurring arsenic in Bangladesh drinking water. *Risk Analysis*, **23**(3):515–528. doi:10.1111/1539-6924.t01-1-00332

Slovic P. 1999 Trust, emotion, sex, politics, and science: surveying the risk-assessment battlefield. *Risk Analysis*, **19**: 689–701.

United Nations. 2015. Department of economic and social affairs: population division. https://esa.un.org/unpd/wup/General/GlossaryDemographicTerms.aspx (accessed 24 September 2016).

United States Environmental Protection Agency. 2015 Human health risk assessment. https://www.epa.gov/risk/human-health-risk-assessment (accessed 31 July 2016).

United States Environmental Protection Agency. 2001 *Risk Assessment Guidance for Superfund (RAGS) Volume III - Part A: Process for Conducting Probabilistic Risk Assessment*, Appendix B/EPA 540-R-02-002, US EPA, Washington, D.C., United States.

Ware J. E. 1987 Standards for validating health measures: definition and content. *Journal of Chronic Disease*, **40**(6): 173–480.

World Health Organization & International Programme on Chemical Safety. 2001 Framework for the Integration of Health and Ecological Risk Assessment. In: *Integrated Risk Assessment*, WHO/IPCS/IRA/01/12, WHO/UNEP/ILP International Programme on Chemical Safety, Geneva, Switzerland.
